# Multimodal analgesia practices for knee and hip arthroplasties in the Netherlands. A prospective observational study from the PAIN OUT registry

**DOI:** 10.1371/journal.pone.0279606

**Published:** 2022-12-22

**Authors:** Marloes Thijssen, Leon Timmerman, Nick J. Koning, Myra Rinia, Jacqueline F. M. van Dijk, Juanita Cheuk-Alam, Kees Olthof, Sjaak Rekker, Monique A. H. Steegers, Regina L. M. van Boekel

**Affiliations:** 1 Department of Anesthesiology and Pain medicine, Antonius Hospital, Nieuwegein, The Netherlands; 2 Department of Anesthesiology and Pain Medicine, Rijnstate Hospital, Arnhem, The Netherlands; 3 Department of Anesthesiology and Pain medicine, University Medical Center Utrecht, Utrecht, The Netherlands; 4 Department of Anesthesiology and Pain Medicine, Erasmus Medical Center, Rotterdam, The Netherlands; 5 Department of Anesthesiology and Pain Medicine, Isala Hospital, Zwolle, The Netherlands; 6 Department of Anesthesiology and Pain Medicine, University Medical Center Groningen, Groningen, The Netherlands; 7 Department of Anesthesiology and Pain Medicine, VU Medical Center, Amsterdam, The Netherlands; 8 Department of Anesthesiology and Pain Medicine, Radboud University Medical Center, Nijmegen, The Netherlands; UCSI: UCSI University, MALAYSIA

## Abstract

**Introduction:**

Different multimodal pain management strategies following total hip arthroplasty(THA) and total knee arthroplasty (TKA) surgery are used in clinical practice. The optimal pain management strategy, however, remains unclear. This study aims to evaluate the differences in perioperative multimodal pain management strategies for THA and TKA in the Netherlands, and studies the associations between patient- and therapy related factors and pain outcomes.

**Methods:**

Data from the Dutch hospitals in the PAIN OUT network were used in this study. Demographic data, pain management strategy including perioperative medication use and anesthetic techniques were recorded and used in a multivariable regression analysis to study the association with maximum pain intensity, the duration of severe pain, pain interference in bed and postoperative nausea.

**Results:**

In 343 hip arthroplasty patients and 301 knee arthroplasty patients in seven hospitals, respectively 28 and 35 different combinations of analgesic regimens were used. The number of different drugs prescribed was not related to postoperative pain intensity. Female sex, younger age and spinal anesthesia were associated with higher postoperative maximum pain scores (Numeric Rating Scale (NRS) > 5). Hip surgery and ketamine use were associated with lower postoperative pain scores. The use of non-steroidal anti-inflammatory drugs (NSAIDs) and gabapentinoids, higher age, higher body mass index (BMI) and male gender were associated with less postoperative nausea (NRS < 3).

**Conclusion:**

In conclusion, our study demonstrated a large diversity of analgesic strategies following total joint arthroplasties in the Netherlands. Although no ideal strategy was identified, the use of NSAIDs, ketamine and dexamethasone were associated with less pain and less side effects.

## Introduction

Total hip arthroplasty (THA) and total knee arthroplasty (TKA) are increasingly performed orthopedic procedures and are expected to increase further in the near future [1]. Previous research demonstrates that severe postoperative pain is common following orthopedic surgery [2]. Effective postoperative pain treatment is essential, because postoperative pain may lead to discomfort, increased complication rate, delayed recovery and chronic pain [3, 4]. Furthermore, patients suffering from acute pain after TKA are more likely to use opioids chronically [5].

Multimodal analgesia is a pain management strategy including two or more different groups of analgesic drugs to improve perioperative analgesia and reduce opioid consumption and the related side effects. Multimodal analgesia regimens are strongly recommended for managing perioperative pain and are considered an important part of Enhanced Recovery After Surgery Programs [6]. Multimodal techniques are commonly used in total joint replacement surgery [7]. The exact pain management protocols vary in current literature and evidence for improving postoperative pain is highly variable. A systematic review by Karlsen et al. included 113 randomized controlled trials (RCT’s), using 37 different treatment interventions. They demonstrated analgesic effects of femoral nerve block, local infiltration analgesia (LIA), non-steroidal anti-inflammatory drugs (NSAID’s) and gabapentinoids, unfortunately it was not possible to indicate an optimal analgesic regimen [8] A study by Maheshwari et al comparing a multimodal approach to placebo after spine surgery did not show any difference in quality of recovery [9].

Although multimodal analgesia is widely studied, treatment strategies vary between hospitals. The optimal combination of analgesic therapies remains unclear. This study aims to 1) evaluate the differences in perioperative pain management practices for THA and TKA in the Netherlands, and 2) to evaluate the associations between patient- and therapy related factors and pain outcomes.

## Methods

The PAIN OUT (www.pain-out.eu) network has been initiated to improve acute postoperative pain management [10]. The network offers a web-based tool to evaluate postoperative pain management practices and to compare findings with other hospitals. Data consist of the validated International Pain Outcomes Questionnaire [11] (IPO-Q) and additional information containing patients demographics, comorbidities, surgical procedures, anesthesia management and pain treatment.

### Design

We conducted a prospective cohort study, using data from the Pain Out registry of seven different hospitals in the Netherlands. Four hospitals were university medical centers, three were regional teaching hospitals. Ethical approval was obtained from the Institutional Review Board (IRB) in each hospital, based on the initial ethical approval of the IRB of the Radboud university medical center (Nijmegen, the Netherlands, authorization number 2017/3541). The methodology is registered with the US National Library of Medicine (ClinicalTrials.gov NCT02083835).

### Patients

Patients who underwent knee or hip replacement surgery were included in the study. Patients were eligible for inclusion if they fulfilled the following criteria: 1) age 18 years or older; 2) on postoperative day one (POD1) they had returned to the ward from the post anesthesia care unit (PACU) for at least six hours; and 3) they consented to take part in the survey. All consecutive eligible patients were informed about the study on the first day after surgery and provided written informed consent. Exclusion criteria were daycare surgery and missing data about anesthesia type or surgery.

### Data collection

In each hospital, study surveyors who were not involved in patients’ care, underwent training for approaching patients. Data collection took place from October 2017 until November 2019. Data were collected on POD1. Data consist of the validated International Pain Outcomes Questionnaire [11] (IPO-Q) and additional information containing patients demographics, comorbidities, surgical procedures, anesthesia management and pain treatment. Data were entered into a web-based, password secure portal where each dataset was given an unique, anonymous code. The PAIN OUT database is hosted and maintained by Jena University Hospital, Germany.

International Classification of Disease procedure (ICD-9) codes were used to select cases of TKA or THA from the Pain Out registry. The database contained all information on baseline characteristics, comorbidities, surgical procedures, anesthetic management, medication used and results from the Pain Out questionnaire [11]. This patient-reported outcome questionnaire, that comprises questions about postoperative pain, interference with activities, side effects and satisfaction about treatment, was completed on POD1.

The following baseline parameters were used in the analyses: sex, body mass index (BMI), comorbidity, substance abuse, preoperative pain and preoperative opioid use. The following analgesic medication or analgesic strategies were registered: acetaminophen, NSAID, ketamine, dexamethasone, gabapentinoids (gabapentin or pregabalin), clonidine, local infiltration analgesia (LIA), locoregional anesthesia and combinations of those.

### Outcome parameters

Our primary outcome parameter was maximum pain intensity (numeric rating scale (NRS), 0 = no pain, up to 10 = most pain imaginable). Secondary parameters were time in severe pain (percentage of the time after surgery), opioid consumption (reported in total morphine equivalents (mg) [12]), nausea (NRS, 0 = no nausea, up to 10 = severe nausea), itching (NRS, 0 = no itching, up to 10 = severe itching), and pain interfering with movement in bed (NRS, 0 = no interference, up to 10 = maximal interference).

### Statistical analysis

In order to evaluate the differences in perioperative pain management practices, descriptive statistics were used to determine the frequencies of the demographic variables and multimodal analgesia strategies. Continuous variables are presented as mean with standard deviation (SD) and compared with the independent samples T-test. Normal distribution was explored using the Shapiro-Wilk test. In case of a non-normal distribution, data are presented as median with inter-quartile range (IQR), and compared using the Kruskal Wallis test.

Binary logistic regression analysis was performed to evaluate whether patient- and treatment characteristics (i.e. sex; comorbidity; substance abuse; preoperative pain; preoperative opioid use; NSAID’s; dexamethasone; ketamine; gabapentinoids; clonidine; LIA; and anesthesia technique) were associated with postoperative pain characteristics (i.e. maximum pain intensity > 5 (NRS), time in severe pain > 20%, nausea score > 3 (NRS) and interference with moving in bed > 3 (NRS)). Cut-off values were defined according to PAINOUT standard analyses criteria [10, 13].

To prevent overfitting of the models, we performed univariable binary logistic regression analyses of all potential factors. Only those factors with a significance level of p ≤ 0.2 were entered into a final multivariable binary logistic regression analysis. To prevent multicollinearity, pairwise correlations between the parameters to be entered into the final models were calculated. Of those with a bivariate correlation of ≥ 0.7 only the parameter with the highest univariable significance level were entered into the final models.

For all statistics, alpha is set at the traditional 0.05 level. All analyses were performed with SPSS (IBM Corp. Released 2013. IBM SPSS Statistics for Windows, Version 25.0. IBM Corporation, Armonk, NY, USA).

## Results

In total, 644 patients were included in this study. Of these, 343 patients underwent THA and 301 patients underwent TKA. Baseline characteristics are shown in Table 1.

**Table 1:** Baseline characteristics.

Characteristics		Hip surgery (n = 343)		Knee surgery (n = 301)	
		Count (%)	Median (IQR*)	Count	Median (IQR)
Sex, male		138 (40)		120 (40)	
Age (years)			69.0 (16.0)		69.0 (12.0)
BMI**			27.0 (6.1)		29.6 (5.9)
Preoperative pain		294 (86)		275 (91)	
Preoperative pain intensity (NRS)			7.0 (2.0)		7.0 (3.0)
Preoperative opioid use		79 (23)		59 (20)	
Renal comorbidity		21 (6)		12 (4)	
Cancer comorbidity		32 (9)		14 (5)	
Psychiatric comorbidity		12 (3)		14 (5)	
Adictive disorder		19 (6)		17 (6)	
Cardiovascular comorbidity		178 (52)		164 (54)	
Gastrointestinal comorbidity		8 (2)		9 (3)	
Respiratory comorbidity		58 (17)		51 (17)	
General Pain syndrome comorbidity		2 (1)		8 (3)	
Diabetes comorbidity		26 (8)		29 (10)	
Revision surgery		25 (7)		26 (9)	
LIA***		8 (2)		232 (77)	
anesthesia technique	General	188 (56)		150 (50)	
	Spinal	150 (44)		140 (47)	
	locoregional + general/sedation	0 (0)		10 (3)	

*interquartile range,

** body mass index,

*** local infiltration analgesia

All patients in our database except for one received acetaminophen, so we did not include acetaminophen in our further analysis. S1 Table shows the most commonly used daily dosage of non-opioid pain medications.

### Hip surgery

Hip surgery was performed in 343 patients. Forty percent of them was male and the median age was 69.0 years. The majority (86%) reported preoperative pain with a median NRS of 7.0 (IQR 2.0). One hundred and eighty-eight participants (56%) received general anesthesia, 150 (44%) spinal anesthesia and no patients reported locoregional anesthesia with or without sedation or general anesthesia.

In patients undergoing hip surgery, 28 different combinations of non-opioid pain medications were used (Table 2). The combinations most commonly used were NSAIDs with ketamine and gabapentinoids, NSAIDs only, NSAIDs with gabapentinoids and ketamine with gabapentinoids.

**Table 2:** Multimodal analgesia strategies hip surgery.

Non-opioid analgisics	n
NSAID, ketamine, gabapentinoid	53
NSAID	43
NSAID, gabapentinoid	34
Ketamine, gabapentinoid	32
NSAID, dexamethasone, ketamine, gabapentinoid	31
No multimodal medication or LIA*	28
Dexamethasone, ketamine, gabapentinoid	22
NSAID, ketamine	14
NSAID, dexamethasone, gabapentinoid	12
Dexamethasone, ketamine	8
Ketamine	8
Gabapentinoid	8
Dexamethasone, gabapentinoid	7
NSAID, clonidine	5
NSAID, ketamine, gabapentinoid, LIA	5
Ketamine, clonidine, gabapentinoid	5
Dexamethasone	4
NSAID, dexamethasone, ketamine	2
Ketamine, clonidine	2
Clonidine	2
NSAID, LIA	1
NSAID, dexamethasone, clonidine	1
NSAID, ketamine, clonidine	1
NSAID, clonidine, gabapentinoid	1
NSAID, gabapentinoid, LIA	1
NSAID, dexamethasone, ketamine, clonidine	1
NSAID, dexamethasone, ketamine, LIA	1
NSAID, dexamethasone, ketamine, clonidine, gabapentinoid	1

* Local infiltration analgesia

Outcome parameters are presented in Table 4. Maximum pain (NRS), time in pain (%), nausea (NRS), itching (NRS) and interference moving in bed (NRS) were not significantly different among the five most commonly used analgesic strategies (S1 Table). Total morphine equivalents were significantly different in these five groups: total morphine equivalents were lowest in the group that received NSAIDs with gabapentinoids (37.0 mg) and highest in the group receiving NSAID with ketamine and gabapentinoids (48.1 mg) (p-value 0.029) (S1 Table).

### Knee surgery

Of the patients undergoing knee surgery 40% was male, median age was 69.0 years. The vast majority (91%) experienced preoperative pain and reported a median NRS of 7.0 (IQR 3.0). One hundred and fifty participants (50%) received general anesthesia, 140 (47%) received spinal anesthesia. Locoregional anesthesia was performed in 10 patients (3%), this included femoral nerve block (n = 9) and epidural anesthesia (n = 1).

We identified 35 different combinations of non-opioid pain medications and LIA for pain treatment in this group. The most commonly used strategies: NSAID with ketamine, gabapentinoids and LIA; NSAID with LIA; NSAID with gabapentinoids and LIA (Table 3).

**Table 3:** Multimodal analgesia strategies knee surgery.

Medicaments	n
NSAID, ketamine, gabapentinoid, LIA*	51
NSAID, LIA	33
NSAID, gabapentinoid, LIA	33
NSAID, ketamine, LIA	16
NSAID, dexamethasone, gabapentinoid, LIA	16
LIA	15
Ketamine, gabapentinoid, LIA	14
NSAID, ketamine, gabapentinoid	12
NSAID, dexamethasone, ketamine, gabapentinoid, LIA	12
Ketamine, gabapentinoid	11
NSAID, gabapentinoid	8
No multimodal medication or LIA	7
NSAID	6
Ketamine	6
NSAID, dexamethasone, ketamine, gabapentinoid	5
Gabapentinoid	5
NSAID, dexamethasone, ketamine, clonidine, gabapentinoid, LIA	4
Dexamethasone, ketamine, gabapentinoid	4
Gabapentinoid, LIA	4
NSAID, dexamethasone, ketamine	3
NSAID, clonidine, gabapentinoid, LIA	3
Dexamethasone, gabapentinoid, LIA	3
Ketamine, clonidine, gabapentinoid, LIA	3
NSAID, ketamine	2
NSAID, clonidine, LIA	2
NSAID, ketamine, clonidine, gabapentinoid, LIA	2
Ketamine, clonidine, LIA	2
NSAID, clonidine	1
NSAID, dexamethasone, gabapentinoid	1
NSAID, dexamethasone, LIA	1
NSAID, ketamine, clonidine, gabapentinoid	1
NSAID, ketamine, clonidine, LIA	1
NSAID, dexamethasone, ketamine, clonidine, gabapentinoid	1
Dexamethasone, ketamine, clonidine, gabapentinoid, LIA	1
Ketamine	1

*Local infiltration analgesia

Postoperative data are listed in Table 4. There were no significant differences in maximum pain (NRS), time in pain (%), nausea (NRS) and pain interfering moving in bed (NRS) among the four most commonly used analgesic strategies (S2 Table). Total morphine equivalents were lowest in the group receiving NSAID with gabapentinoids and LIA, and highest among the group receiving NSAID with ketamine and LIA (p-value 0.039). Itch was most commonly reported in the group receiving NSAID and LIA, with a mean NRS 0.9 (p-value 0.043) (S2 Table).

**Table 4:** Postoperative pain and side effects.

Variables *	Hip surgery	Knee surgery
	Median (IQR**)	Median (IQR)
Maximum pain	6.0 (4.0)	7.0 (3.0)
time in severe pain (%)	30% (40)	40% (40)
Postoperative opioids (mg) ***	30.0 (22.5)	37.5 (27.8)
Nausea	0.0 (3.0)	1.0 (5.0)
Itching	0.0 (0.0)	0.0 (0.0)
Pain interfering moving in bed	5.0 (5.0)	6.0 (4.0)
Pain interfering moving out of bed	4.0 (5.0)	6.0 (4.0)

* NRS if not stated otherwise,

** Interquartile range,

*** total morphine equivalents

### Number of drug classes

**Fig 1:**
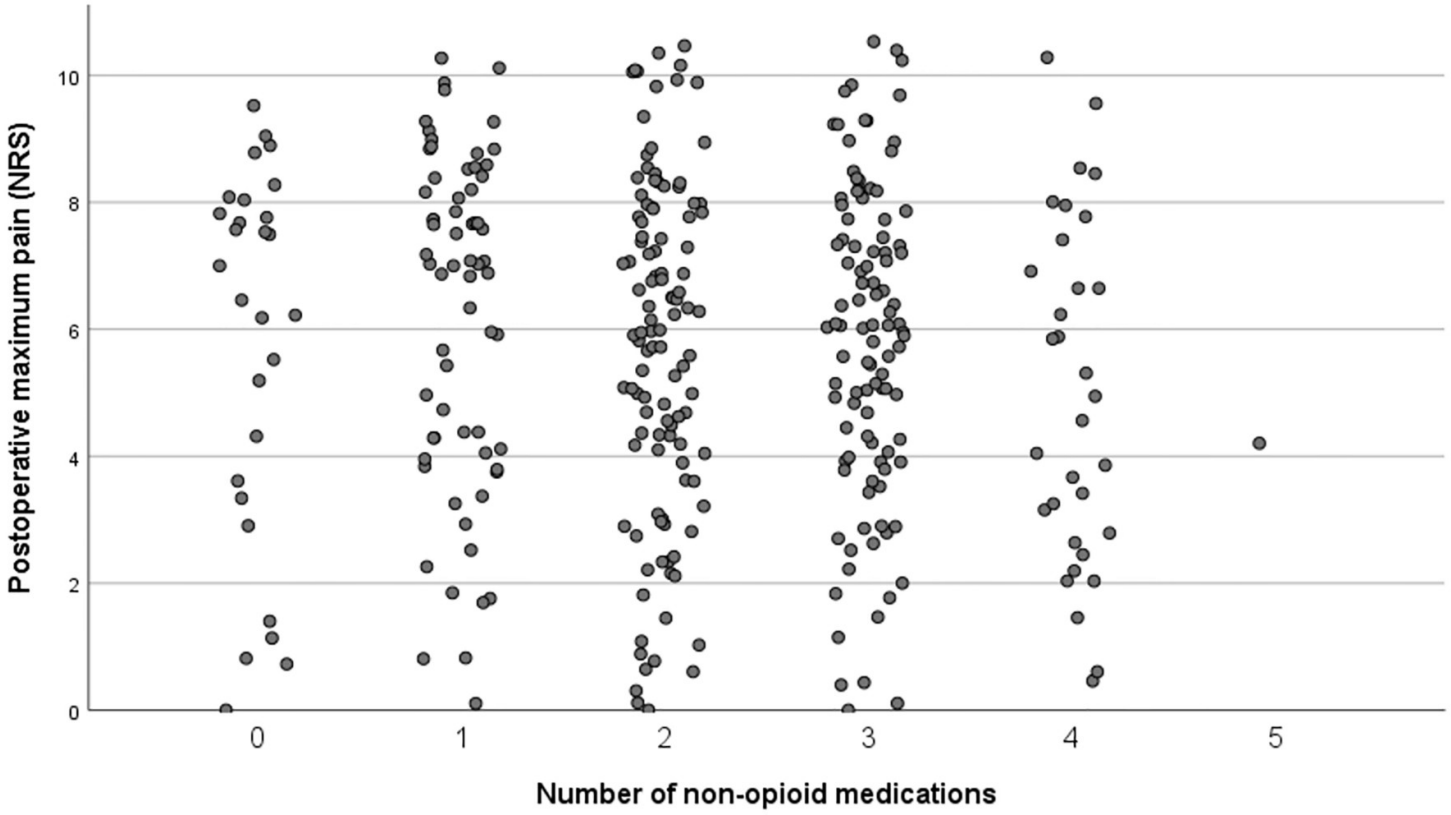
Postoperative maximum pain (NRS) after THA by number of non-opioid medication.

**Fig 2:**
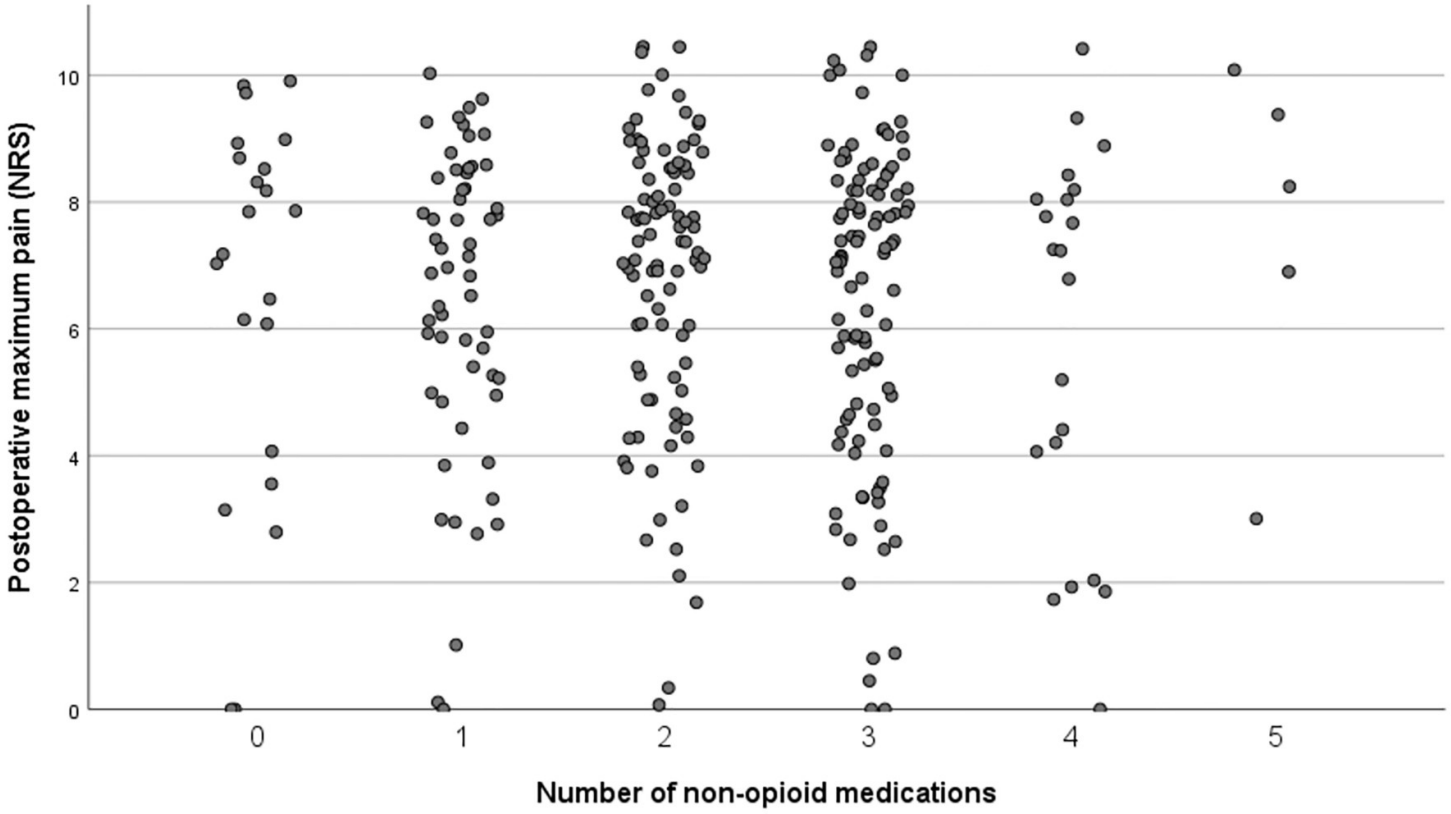
Postoperative maximum pain (NRS) after TKA by number of non-opioid medication.

Median postoperative maximum pain (NRS) was not statistically different when using more drugs (p = 0,328). Median postoperative pain did not differ when using more medication in patients undergoing hip surgery (p = 0.229) and knee surgery (p = 0.656) (Figs 1 and 2).

### Multivariate logistic regression analysis

Table 5 shows the multivariate logistic regression analysis for maximum postoperative pain, time in severe pain, pain when moving in bed and nausea in the total study population. Female sex, younger age and spinal anesthesia were associated with postoperative maximum pain > 5 (NRS). Hip surgery and ketamine use were associated with less postoperative pain. Knee surgery, female sex, younger age and cardiovascular comorbidity were associated with time in severe pain > 20%. Knee surgery, female sex, younger age, lower BMI were associated with nausea > 3 (NRS), whereas the use of NSAIDs and gabapentinoids was associated with less postoperative nausea. S3 and S4 Tables show the multivariate logistic regression analysis for postoperative pain and nausea in hip- and knee surgery, respectively.

**Table 5:** Multivariate logistic regression analysis.

Variables	Exp (B)	95% CI	p-value
**Maximum pain > 5 (NRS)** *			
Sex (male)	0.50	0.36–0.71	0.000
Age (years)	0.98	0.96–0.99	0.004
Hip surgery	0.58	0.41–0.82	0.002
Spinal anesthesia	1.45	1.03–2.05	0.032
Ketamine	0.63	0.45–0.89	0.008
Clonidine	2.05	0.96–4.37	0.065
**Time in severe pain > 20%** **			
Knee surgery	2.47	1.75–3.49	0.000
Sex (male)	0.54	0.38–0.76	0.000
Age (years)	0.98	0.96–0.99	0.003
cardiovascular comorbidity	1.50	1.05–2.14	0.027
Diabetes	1.84	0.94–3.63	0.076
**pain moving in bed > 3** ***			
Sex (male)	0.65	0.45–0.93	0.018
Age (years)	0.98	0.96–0.99	0.005
Psychiatric comorbidity	2.95	0.85–10.20	0.087
Knee surgery	1.62	1.13–2.33	0.009
NSAID	0.56	0.38–0.84	0.005
Dexamethasone	0.67	0.45–1.02	0.061
Ketamine	0.63	0.43–0.91	0.013
**Nausea > 3** ****			
Knee surgery	2.27	1.51–3.41	0.000
Sex (male)	0.36	0.23–0.54	0.000
Age (years)	0.98	0.96–1.00	0.022
BMI	0.93	0.90–0.97	0.000
Preoperative pain	1.80	0.95–3.42	0.074
Gabapentinoids	0.51	0.34–0.77	0.002
General pain syndrome	3.35	0.83–13.60	0.090
NSAID	0.53	0.36–0.79	0.002

* Variables entered on step 1: Sex, Age, BMI, General Pain syndrome, Psychiatric comorbidity, Preoperative pain, Preoperative opioid use, Hip surgery, Knee surgery, Spinal anesthesia, LIA, Dexamethasone, Ketamine, Clonidine.

** Variables entered on step 1: Spinal anesthesia, Hip surgery, Knee surgery, Sex, Age, BMI, Cardiovascular comorbidity, Diabetes comorbidity, Respiratory comorbidity, Preoperative pain, Preoperative opioid use, LIA, Dexamethasone, Gabapentinoids.

*** Variables entered on step 1: Sex, Age, BMI, Psychiatric comorbidity, Preoperative pain, Hip surgery, Knee surgery, NSAID, Dexamethasone, Ketamine.

**** Variables entered on step 1: Spinal anesthesia, Hip surgery, Knee surgery, Sex, Age, BMI, Preoperative pain, LIA, Dexamethasone, Gabapentinoids, General Pain syndrome, General anesthesia, NSAID, Ketamine.

### Post-hoc analysis

We performed a post-hoc analysis comparing 43 patients receiving acetaminophen and NSAIDs only did not differ from 174 patients receiving acetaminophen and NSAID and additional pain medications for THA (median maximum pain NRS 7.0 vs 6.0 (p = 0.754), and median time in severe pain 30% vs 30% (p = 0.205)).

## Discussion

The aim of this study was to evaluate the differences in perioperative pain management practices for THA and TKA in different hospitals in the Netherlands, and to evaluate the associations between patient- and therapy related factors and pain outcomes. The inclusion of multiple hospitals in the Netherlands provided a clear picture of the different strategies used for treating postoperative pain across the country. We used the PAIN-Out questionnaire, which is internationally validated [11]. Our results show that there is a large variety in multimodal analgesia strategies for patients undergoing total hip or knee replacement in the Netherlands. Despite significant attention for optimal pain control, many patients reported severe postoperative pain. Median postoperative pain intensities were high following THA and even higher in TKA. These data are more or less in line with data reported in studies in Portugal [14], Germany [15, 16] and a large international multicenter cohort study [17].

We did not find a significant difference in pain scores between the most commonly used multimodal analgesia strategies. The number of different drugs prescribed was not related to postoperative pain intensity. We found some statistically differences in total morphine equivalents between the multimodal strategies used. Unfortunately the study design does not allow us to confirm causality.

Recently, the new PROSPECT guideline for total knee arthroplasty recommends a combination of acetaminophen, NSAID and dexamethasone, together with LIA and single shot adductor canal block for managing postoperative pain. There was conflicting or inconsistent evidence for other intravenous analgesics like gabapentinoids, ketamine and dexmedetomidine [18]. In line with these outcomes, a recent RCT did not find benefits of adding dexmedetomidine, ketamine, repeated doses dexamethasone and additive nerve blocks to a multimodal regimen containing LIA, intrathecal morphine, single shot adductor canal block and dexamethasone [19]. According to the PROSPECT guideline for total hip arthroplasty, a combination of acetaminophen and a non-steroidal anti-inflammatory drug or a cyclo-oxygenase-2-selective inhibitor and intravenous dexamethasone improve postoperative pain [20]. There was limited or no evidence found for all other approaches. These results were confirmed in a post-hoc analysis of our data, in which pain intensity between patients receiving acetaminophen and NSAIDs only did not differ from patients receiving additional pain medications for THA. Therefore, a lot of different strategies are used to manage postoperative pain. Choices are not solely based on optimal pain reduction. Rapid recovery, early mobilization and early discharge from hospital might be conflicting treatment goals that play an important role as well in choosing a pain management strategy. Improved preoperative risk stratification might enable individualized postoperative pain therapy tailored to the needs of the patients. Although pharmacological postoperative pain management has been studied extensively, emerging non-pharmacological therapies such as education and psychological preparation for surgery are encouraging and deserve additional scientific attention [21].

We found several associations that are well known to predict postoperative pain. Lower age and female gender are associated with higher postoperative pain intensities, and a psychiatric comorbidity was related to pain during mobilization. Furthermore, some relations were found regarding pharmacotherapy. The use of ketamine and the use of NSAIDs were related to less pain during movement following knee surgery. The use of NSAIDs was associated with less nausea following THA and TKA and gabapentin use was associated with less nausea following TKA. These effects might be explained by the opioid sparing properties of these analgesics. Dexamethasone was associated with lower pain intensities and less nausea after THA. Unexpectedly, gabapentinoids were positively related to longer time in pain after TKA. Gabapentinoids were generally provided as part of opioid free rapid recovery programs in which functional recovery and early discharge were the primary goals of therapy. However, contribution of patient and surgery-related factors cannot be ruled out. García-López and colleagues demonstrated in a recent study the following factors associated with moderate to severe postoperative pain after TKA: chronic preoperative pain, general anesthesia, opioid use before or after surgery. There were no protective factors observed [17]. This is partly in line with our results, demonstrating preoperative pain and female gender were associated with severe postoperative pain. However, in our database spinal anesthesia instead of general anesthesia was a risk factor for high pain intensity following surgery. This might be explained by the delayed administration of other analgesic therapy during the first hours after surgery causing a rebound effect and increased pain after the sensory block wears off.

In this database, locoregional anesthesia was rarely used. Previous research shows an increasing popularity of peripheral nerve blocks, however it is still used in only a minority of patients [22]. Classically, femoral nerve block has been performed for analgesia after TKA, resulting in improved analgesia. Because of motor weakness, delaying mobilization after surgery, femoral nerve block is not often performed anymore. Other peripheral nerve blocks, including the adductor canal block and interspace between the popliteal artery and posterior capsule of the knee (IPACK) block, might reduce postoperative opioid use and postoperative pain scores and are currently recommended for pain management following TKA [18, 23]. Few studies have been evaluating the use of pericapsular nerve block nerve group (PENG) block for THA. This block results in less pain when compared to no block, however there is only a short term reduction of pain when compared to intra-articular local anesthetic injection [24, 25].

One of the limitations of this study is the fact that postoperative pain was only evaluated once and only on POD 1. Answers could be influenced, not only by the level of pain while filling in the questionnaire, but also by the timing of the analgesics administered by the wards relative to the timing of the questionnaire. Due to the cross-sectional design of this study causality cannot be confirmed. We managed to create a large database containing the different strategies in our country, however, the data contained a lot of different combinations of multimodal drugs. Due to this large diversity of pain strategies, we were only able to study the individual relation between specific drugs and therapy outcome in our regression analysis. To evaluate the effects of specific combinations of analgesics, prospective comparative studies or a large sample size are needed.

In conclusion, our study demonstrated a large diversity of analgesic strategies following total joint arthroplasties in the Netherlands. The use of more drug classes does not show lower postoperative pain scores. Although no ideal strategy was identified, the use of NSAID’s, ketamine and dexamethasone were independently associated with less pain and less side effects.

## Supporting information

S1 Table Most commonly used daily dosage of non-opioid pain medications. (XLSX)

S2 Table Multimodal strategy hip surgery. *NRS if not stated otherwise **Kruskal Wallis test.
(XLSX)

S3 Table Multimodal strategy knee surgery. *NRS if not stated otherwise; **Kruskal Wallis test.
(XLSX)

S4 Table Multivariate logistic regression analysis, hip surgery. * Variables entered on step 1: Sex, Age, BMI, Psychiatric comorbidity, Preoperative opioid use, Dexamethasone, Ketamine, Clonidine. ** Variables entered on step 1: Sex, Age, BMI, Diabetes, Respiratory comorbidity, Spinal anesthesia, Locoregional anesthesia, LIA, Dexamethasone. *** Variables entered on step 1: Sex, Age, BMI, Psychiatric comorbidity, LIA, NSAID, Dexamethasone. **** Variables entered on step 1: Sex, Age, BMI, Addictive disorder, Spinal anesthesia, General anesthesia, Locoregional anesthesia, NSAID, Dexamethasone, Gabapentinoids.
(XLSX)

S5 Table Multivariate logistic regression analysis, knee surgery. * Variables entered on step 1: Sex, Preoperative pain, Preoperative opioid use, General anesthesia, Ketamine. ** Variables entered on step 1: Sex, Cardiovascular comorbidity, Preoperative opioid use, Gabapentinoids. *** Variables entered on step 1: Age, NSAID, Ketamine. **** Variables entered on step 1: Sex, BMI, Cardiovascular comorbidity, General Pain syndrome, NSAID, Ketamine, Gabapentinoids, Clonidine.
(XLSX)

S1 File (XLSX)
