# Correction: Multimodal analgesia practices for knee and hip arthroplasties in the Netherlands. A prospective observational study from the PAIN OUT registry

**DOI:** 10.1371/journal.pone.0331852

**Published:** 2025-09-05

**Authors:** Marloes Thijssen, Leon Timmerman, Nick J. Koning, Myra Rinia, Jacqueline F.M. van Dijk, Mienke Rijsdijk, Juanita Cheuk-Alam, Kees Olthof, Sjaak Rekker, Monique A.H. Steegers, Regina L.M. van Boekel

Mienke Rijsdijk should be included in the author byline. Mienke Rijsdijk should be listed as the sixth author, and his affiliation is 3: Department of Anesthesiology and Pain medicine, University Medical Center Utrecht, Utrecht, the Netherlands. The contributions of this author are as follows: Conceptualization, Data Curation, Methodology and Writing – Review & Editing.

The correct citation is: Thijssen M, Timmerman L, Koning NJ, Rinia M, van Dijk JFM, Rijsdijk M, et al. (2022) Multimodal analgesia practices for knee and hip arthroplasties in the Netherlands. A prospective observational study from the PAIN OUT registry. PLoS ONE 17(12): e0279606. https://doi.org/10.1371/journal.pone.0279606
